# Topographic and Anatomical Landmarks of Key Points in Embryologically Guided Surgery for Locally Advanced Gastric Cancer Using Computer-Assisted 3D Navigation

**DOI:** 10.3390/jcm14176282

**Published:** 2025-09-05

**Authors:** Tatiana Khorobrykh, Vadim Agadzhanov, Anton Grachalov, Ivan Ivashov, Alexey Spartak, Artem Romanovskii, Yaroslav Drach, Daniil Kharkov

**Affiliations:** 1 Department of Faculty Surgery No. 2, Named after G.I. Lukomsky, Federal State Autonomous Educational Institution of Higher Education I.M. Sechenov First Moscow State Medical University of the Ministry of Health of the Russian Federation (Sechenov University), 8-2 Trubetskaya Str., 119991 Moscow, Russia; horobryh68@list.ru (T.K.); agadjanov@mail.ru (V.A.); grachalov98@mail.ru (A.G.); alspartak@yandex.ru (A.S.); romanovskiy_a_a1@student.sechenov.ru (A.R.); kharkov_d_i@student.sechenov.ru (D.K.); 2 Department Biomedical Technical Systems of Faculty Biomedical Engineering, Federal State Autonomous Educational Institution of Higher Education Bauman Moscow State Technical University, 2-ya 5/1, Baumanskaya Str., 105005 Moscow, Russia; jaroslav.ing.02@gmail.com

**Keywords:** locally advanced gastric cancer, laparoscopic gastrectomy, mesogastric layer, embryological dissection plane, topographic-anatomical navigation, 3D surgical planning

## Abstract

**Background/Objectives:** Gastric cancer remains a leading cause of cancer-related mortality, with over 50% of cases diagnosed at a locally advanced or metastatic stage. High-quality surgical resection within the embryological mesogastric layer is critical for achieving optimal oncological outcomes but is often complicated by anatomical distortion in advanced tumors. This study aimed to develop and validate a system of topographic and anatomical navigation landmarks for embryologically guided laparoscopic gastrectomy, leveraging 3D modeling to enhance precision and safety. **Methods:** A single-center study was conducted, analyzing 78 patients undergoing emergency laparoscopic gastrectomy for locally advanced gastric cancer. Preoperative 3D models were generated from CT data annotations to map the stomach, tumor, vascular structures, and mesogastric adipose tissue. Thirty biomodels were used to refine dissection techniques. Surgical procedures adhered to embryological principles, with lymphadenectomy guided by predefined landmarks. Histopathological validation assessed resection margins and tumor infiltration in resected specimens. Statistical analysis compared outcomes between patients with and without 3D planning. **Results:** The 3D models demonstrated 100% concordance with intraoperative vascular anatomy. Radiologically dense adipose tissue, resected as potentially tumor-infiltrated, showed histopathological invasion in 74% of cases. R0 resection was achieved in 74.4% of patients. Operative time decreased from 300 to 250 min after technical optimization, with a 7.7% conversion rate (primarily due to vascular injury or tumor fixation). Postoperative mortality was 5.1%, attributed to comorbidities. Patients with 3D planning had significantly higher lymph node yields (*p* < 0.00001) and R0 rates (*p* = 0.045). **Conclusions:** The integration of embryologically based topographic landmarks and 3D navigation improves the safety and standardization of laparoscopic gastrectomy for locally advanced gastric cancer. This approach enhances oncological radicality, reduces operative time, and mitigates risks in anatomically distorted fields. Further validation in larger cohorts is warranted.

## 1. Introduction

Gastric cancer remains a major global health burden, ranking as the fourth leading cause of cancer-related mortality and fifth in overall cancer incidence. Despite recent declines in morbidity and mortality rates, epidemiological data from 2020 reported over 1 million new cases worldwide with 769,000 deaths, underscoring its significant impact. Prognosis is critically dependent on disease stage at diagnosis, with more than half of patients presenting with locally advanced or metastatic disease—clinical scenarios associated with particularly poor outcomes [1].

The cornerstone of effective treatment lies in high-quality surgical intervention [2]. Modern surgical techniques have undergone a transformative evolution through revolutionary insights into abdominal embryogenesis and spatial anatomy. This paradigm shift has particularly enhanced our understanding of three fundamental aspects: mesenteric embryological development, fascial plane ontogeny, and compartmental tumor spread patterns [3,4,5].

Optimal oncological outcomes are achieved through R0 resection, requiring complete tumor removal within the embryonic mesenteric layer along with its associated lymphatic basins, vascular networks, and potentially infiltrated mesenchymal tissues. The oncological adequacy of surgical specimens—assessed through margin status, lymph node yield, and mesofascial integrity—has emerged as a critical determinant of disease-free survival. This recognition has driven renewed focus on developmental anatomy and compartmental resection techniques [2,6,7].

The principle of embryologically guided gastric cancer surgery involves precise alignment of resection planes with the boundaries of the embryonic layer derived from the primitive dorsal mesentery (future mesogastrium). Accurate identification of mesogastric layer margins is essential for performing oncologically sound resections, including the determination of obligatory transection points needed to preserve the pancreas and spleen—organs sharing this embryological origin. Successful dissection requires restoration of the dorsal mesentery’s original pre-rotation architecture while maintaining appropriate lymphadenectomy extent [2,6,7].

Laparoscopic approaches offer distinct advantages through superior anatomical visualization, which enhances surgical safety, facilitates thorough lymph node dissection, reduces procedural trauma and blood loss, and decreases postoperative complications [8,9]. However, technical challenges emerge in locally advanced cases where tumor-induced anatomical distortion is compounded by the inherent lack of tactile feedback in endoscopic surgery, particularly when operating near major vascular structures within compromised anatomical planes.

Embryological studies confirm that complete gastric resection within the mesogastric plane necessarily requires pancreaticosplenectomy [8,9], making mesogastric layer resection a carefully balanced surgical compromise. This challenge is particularly pronounced in locally advanced disease where tumor infiltration causes mesogastric layer deformation and invasion. Current advancements addressing these difficulties include intraoperative medical tracers (ICG), radiopharmaceutical administration with intraoperative CT, and emerging artificial intelligence applications [10,11,12].

Radiological assessment presents its own challenges, with CT imaging of locally advanced gastric cancer demonstrating increased perigastric adipose tissue density manifesting as coarse or fine linear stranding and nodular formations. These changes often result in Hounsfield unit measurements approaching tumor density values, significantly obscuring the demarcation between gastric wall boundaries and adjacent tissues [2,5,6,13,14,15].

Advanced imaging processing through segmentation and three-dimensional reconstruction enables visualization of lymphatic drainage pathways and generation of patient-specific tumor models for preoperative planning.

Critical anatomical landmarks demanding particular surgical attention include the pancreatic head/isthmus region and splenic hilum—areas where obligatory mesogastric layer transection adjacent to major vasculature is required. At these sites, surgeons must carefully balance three competing priorities: extending dissection beyond the mesogastric plane, preserving colonic vascular supply, and maintaining adherence to oncological radical resection principles. Precise alignment of surgical resection planes with embryological mesogastric layer boundaries constitutes a fundamental requirement for complete (D2) lymphadenectomy in all foregut-derived malignancies, with particular significance in gastric cancer.

### Study Aim and Objectives

This study aims to systematically delineate the operative sequence of laparoscopic gastrectomy within embryologically defined mesogastric planes for locally advanced gastric cancer, establishing reproducible anatomical landmarks to guide oncologically radical dissection while optimizing surgical outcomes.

## 2. Materials and Methods

### 2.1. Study Design

The present work represents a single-center prospective and retrospective study performed at a tertiary surgical department (Sechenov University). All procedures followed evidence-based oncological principles while accounting for individual anatomical variations and applying embryologically guided dissection techniques as first proposed by Congdon (1942) in his fascial fusion theory [16,17,18,19,20,21,22,23,24].

The study employed 30 anatomical biomodels to systematically investigate the boundaries of the mesogastric layer, identify critical points of obligatory transection, and evaluate the feasibility of safe surgical manipulation within this anatomical plane. These biomodels served as an essential training platform for mastering the complex spatial relationships between embryological fascial planes and adjacent vascular structures prior to clinical application.

A total of 78 patients underwent emergency surgical intervention due to life-threatening complications of their underlying disease. The indications for surgery included (1) tumor-induced stenosis of the cardia and gastric outlet (41 patients) and (2) recurrent tumor bleeding (22 patients). Nine patients presented with combined proximal/distal stenosis accompanied by recurrent hemorrhagic episodes. The mean patient age was 60 years (range: 58–62), with a male-to-female ratio of 2.1:1. Most patients had documented cardiovascular comorbidities.

**Inclusion Criteria:**

1. Age ≥ 18 years;
2. ECOG performance status ≤ 2;
3. Histologically confirmed gastric adenocarcinoma confirmed by preoperative esophagogastroduodenoscopy with biopsy;
4. PET-CT approved by the multidisciplinary tumor board;
5. Written informed consent obtained.

**Exclusion Criteria:**

1. Diffuse peritoneal carcinomatosis (parietal or visceral);
2. Synchronous malignant tumors;
3. ASA > 3;
4. Pregnancy and breast-feeding patients;
5. Severe mental disorders.

To address perioperative nutritional deficits, patients received LEOVIT ONCO enteral nutrition (domestically produced in Russia), which has demonstrated clinical efficacy as part of combination therapy for cancer patients with complicated forms of locally advanced gastric cancer [25]. The patient characteristics are summarized in Table 1.

As an adjunctive navigation tool, our multidisciplinary team comprising radiologists and 3D imaging specialists performed advanced preoperative modeling for 25 patients. This comprehensive 3D reconstruction integrated the following: (1) gastric and tumor volumetry, (2) celiac axis vascular mapping with portal venous system relations, (3) densitometric analysis of paracardiac adipose tissue (designated as the mesogastric compartment). The mesogastric layer assessment protocol was based on quantitative imaging biomarkers: structural alterations in paracardiac fat (involving Japanese Classification lymph node stations 1, 7, and 9) manifested as characteristic stranding patterns (linear densities > −30 Hounsfield units) and contrast enhancement differentials (malignant nodes > 100 HU vs. benign < 60 HU).

For proximal/cardia tumors, we established significant correlations between fat density gradients (ΔHU ≥ 40) and histologically confirmed mesogastric invasion (*p* = 0.007), while distal tumors demonstrated a predilection for infrapyloric nodal conglomerates (station 6) with 89% preoperative identification accuracy. The modeling workflow incorporated multiphase CT segmentation, computational fluid dynamics for vascular simulation, and patient-specific 3D printing, achieving 94% sensitivity for predicting R0 resection boundaries when validated against surgical specimens.

**Figure 1:**
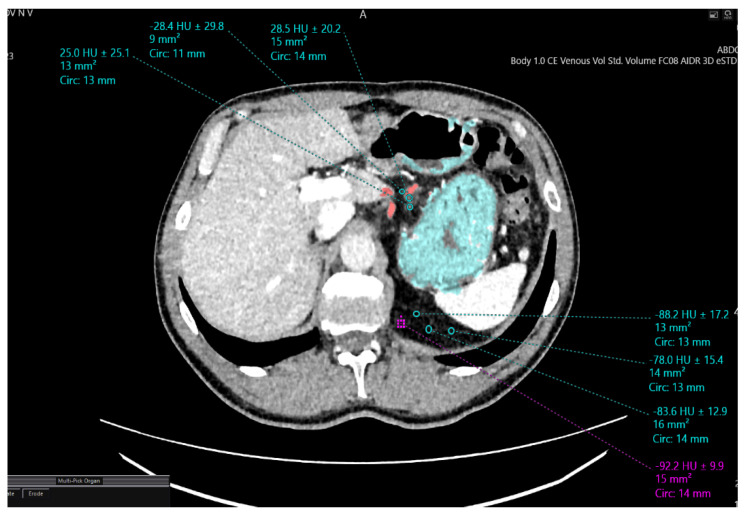
Measurement of adipose tissue density in the venous phase.

**Figure 2:**
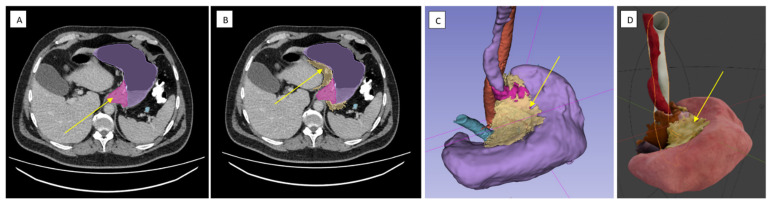
Stages of 3D reconstruction. (**A**) Abdominal CT scan with demarcation of gastric tumor (arrow) and unaffected stomach tissue; (**B**) abdominal CT scan showing annotated mesogastric layer (arrow); (**C**) 3D reconstruction of mesogastric layer (arrow) and tumor using 3D Slicer software; (**D**) volume-rendered (VR) reconstruction model (mesogastric layer is indicated with arrow).

The 3D reconstruction protocol was implemented as follows: Venous-phase CT scans were utilized to manually annotate arterial structures within the celiac trunk basin, including the left gastric artery. Pathological fat infiltration boundaries along the left gastric artery corridor were then demarcated by three board-certified radiologists (each with >10 years of specialized experience), with consensus-defined subjective margins based on these key criteria: density > −30 HU, irregular borders, and stranding patterns (Figure 1 and Figure 2).

Standardized landmarking was similarly performed for the greater omentum and retroperitoneal fat compartments, with normal reference tissue (density < −50 HU, homogeneous architecture) serving as anatomical controls (Table 2). All reconstructions were processed using 3D Slicer software (version 5.6.2 or earlier). Visualization of individual segmentations—including the tumor, stomach, veins, arteries, affected lymph nodes, and infiltrated adipose tissue—was performed using 3D Slicer’s built-in tools, with surface smoothing adjustments applied. The final 3D model was assembled on a single scene by sequentially adding all segmented structures while preserving the spatial coordinates from the original DICOM file.

Prior to determining the surgical treatment strategy, all cases were presented and discussed at a multidisciplinary oncology consultation. The inclusion criteria for patients were as follows: absence of widespread carcinomatosis of the parietal and visceral peritoneum, absence of high esophageal tumor involvement (above 4 cm), absence of severe abdominal adhesions, and an overall ECOG performance status of 1–2.

### 2.2. Statistical Analysis

Statistical data processing was performed to assess the significance of differences between groups of patients who underwent preoperative 3D planning and those who did not. The sample size consisted of 78 cases, necessitating the use of nonparametric methods, which are robust to deviations from a normal distribution and applicable to relatively small datasets.

For comparing quantitative variables between two independent groups, the Mann–Whitney U test was used as an alternative to the *t*-test when a normal distribution could not be guaranteed. This test was applied to evaluate differences in the following parameters: mean duration of surgery, number of removed lymph nodes, intraoperative blood loss, postoperative mortality rate, and frequency of conversion to laparotomy.

To assess differences in variability (variances) of parameters between groups, the Brown–Forsythe test was employed, recommended for small samples and non-normal distributions as an alternative to the Levene test.

Categorical variables, specifically the radicality of surgical intervention (R0 versus R1), were analyzed using Fisher’s exact test, which is the most reliable method for comparing frequencies in 2 × 2 contingency tables with small cell counts.

Data analysis was conducted using the Python 3.11 programming language in the Jupyter Notebook environment. Statistical computations were performed using the Pandas 2.2.1, NumPy 1.26.4, and SciPy 1.12.0 libraries.

### 2.3. Technical Aspects of the Surgical Procedures

The extent of surgical intervention was determined by tumor location. For total and subtotal gastric involvement or tumors in the upper third of the stomach body, we performed gastrectomy with resection of the abdominal esophagus. For tumors in the lower third of the stomach body and antrum, distal subtotal gastrectomy was performed. When the tumor was located in the middle third of the stomach body and adequate resection margins could be achieved, we performed distal near-total gastrectomy; otherwise, total gastrectomy was undertaken. To maintain blood supply to the remnant stomach, one to two short gastric arteries and the so-called posterior gastric artery were preserved. For localized tumors of the gastric cardia, the procedure of choice was proximal gastrectomy with resection of the abdominal and lower thoracic esophagus. In distal resections, Billroth I reconstruction was preferred due to its functional advantages. Billroth II reconstruction was indicated when tumor invasion extended to the pylorus and duodenum or when there was a lymph node conglomerate in the pyloric region. The extent of recommended lymphadenectomy for gastric cancer was tailored to the type of surgical procedure performed, in accordance with current clinical guidelines [19].

The clinic performs no fewer than 150 gastric surgeries annually. The developed navigation system is based on classical anatomical data, a review of global surgical experience in gastric cancer treatment, and our institutional expertise in both open and laparoscopic approaches.

To evaluate the efficacy of this navigation system, we analyzed 78 video-recorded laparoscopic procedures performed for complicated locally advanced gastric cancer across various tumor locations.

#### 2.3.1. Surgical Dissection Planes and Landmarks: Critical Steps of Gastrectomy in the Embryonic Plane

To facilitate identification of mesogastric layer boundaries, particularly in cases of tumor tissue infiltration, we established imaginary dissection planes traversing stable anatomical landmarks. These planes constitute the first critical step in embryonic-plane gastrectomy.

The sagittal plane traversing the round ligament of the liver—which projectively aligns with the course of the superior mesenteric vein—demarcates lymphatic stations 5 and 6, along with vascular structures located to the right that pertain to the pancreatic head. This plane simultaneously represents the fusion zone of ventral and dorsal embryonic mesenteric layers, which we necessarily transect during dissection.

**Figure 3:**
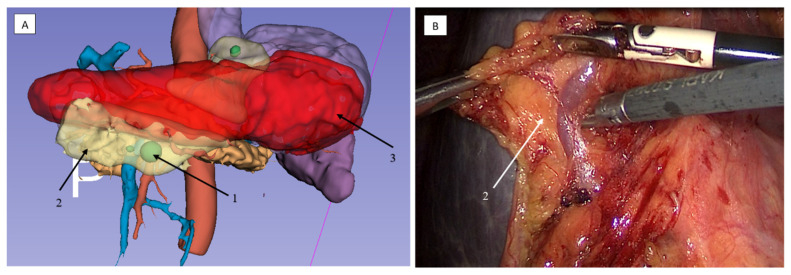
(**A**) 3D reconstruction model; (**B**) intraoperative photograph. Enlarged pyloric lymph nodes (station 6) (1) with tumor-infiltrated adipose tissue (2) and primary gastric carcinoma (3). Posteriorly, tributaries of Henle’s trunk are visualized.

The right colic vein serves as the inferior horizontal boundary for adequate mobilization of the transverse mesocolon. Proceeding upward from its confluence with Henle’s trunk, we identify the right gastroepiploic vein (Figure 3).

**Figure 4:**
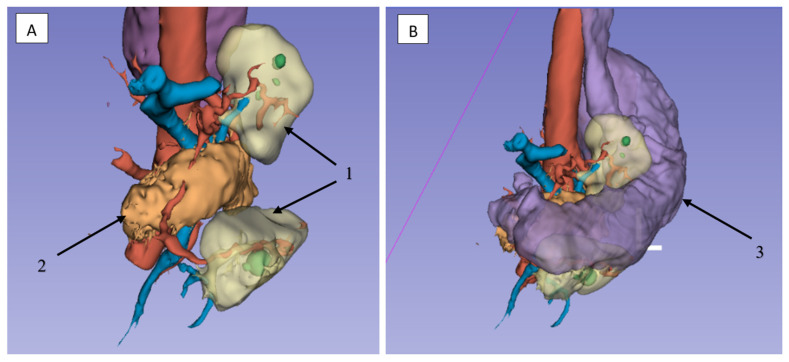
Compromised mesogastrium without (**A**) and with (**B**) the stomach to demonstrate anatomical landmarks for lymphadenectomy in the pancreatic head region (1—mesogastrium, 2—pancreas, 3—stomach).

Thus, advancing within the embryonic plane, we separate the mesocolon from the anterior leaf of the mesogastric layer and the pancreatic head embedded here during embryogenesis (Figure 4). The posterior boundary of adequate lymphadenectomy is formed by the supramesocolic fascia covering the pancreatic head. Its inferior margin is defined by the base of the transverse mesocolon root, where the right colic vein traverses.

**Figure 5:**
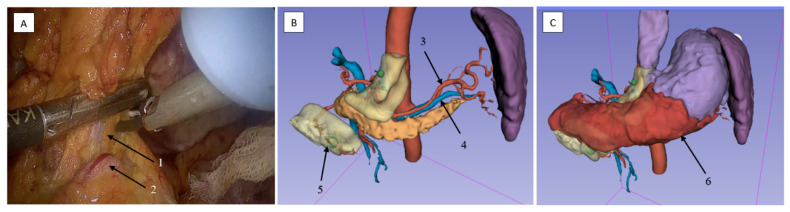
(**A**) Intraoperative photo; (**B**,**C**) 3D reconstruction of the surgical area. The course of the splenic artery (2), (3) and vein (1), (4) along the upper edge of the pancreas, where the regional lymph nodes are located; adipose tissue with regional lymph nodes (5); tumor involving the stomach (6).

The horizontal plane following the superior pancreatic border defines the mesogastric layer to be resected during lymphadenectomy of stations 8, 9, and 11p (Figure 5).

Identification of key landmarks within the horizontal plane—including the celiac trunk elements and left gastroepiploic artery—enables anterior-to-posterior dissection through the mesogastric layer toward the retroperitoneal space without penetrating deep retroperitoneal structures, violating the pancreatic parenchyma, endangering the splenic vein, or disrupting Gerota’s fascia. This approach permits safe medial–lateral, lateral–medial, and superior dissection up to the diaphragmatic crura.

The left gastroepiploic artery marks another fusion point between the mesogastric and mesocolic layers of the embryonic mesentery. Lateral to this artery, the omental bursa cavity is no longer present [2].

**Figure 6:**
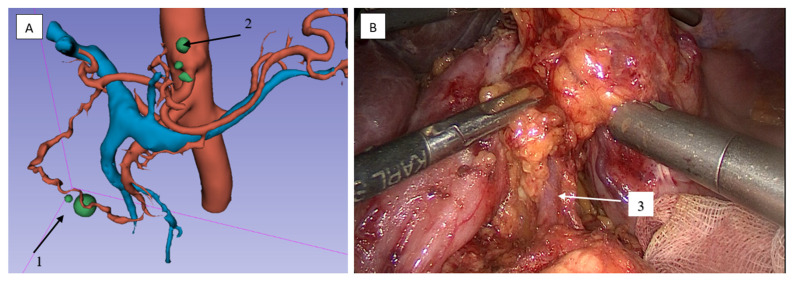
(**A**) 3D reconstruction of angioarchitecture and lymph nodes. (**B**) Intraoperative photo. Lymph nodes along the right gastroepiploic artery (1) and along the celiac trunk (2); left gastric artery (3).

Further mediolateral dissection transitions into the intermediate layer, increasing risks of splenic and splenic vein injury. The splenic hilum mesolayer primarily serves as a mesenchymal conduit for the spleen rather than the stomach, permitting its preservation without compromising oncologic principles. The left gastroepiploic artery region constitutes the second critical landmark for embryonic-plane gastrectomy (Figure 6).

Further dissection in this zone proceeds along the posterior circumference of the splenic artery toward the diaphragmatic crura. Particular attention must be paid to anatomical variations of the left gastroepiploic artery, which may originate not only from the splenic artery but also from the inferior polar splenic artery [2]. By dividing the splenocolic ligament of Morgenstern, we separate the fused mesogastric and mesocolic layers resulting from embryonic rotation. This technical maneuver also exposes the splenic inferior pole, providing new landmarks for transecting the short gastric vessels.

#### 2.3.2. Lymphadenectomy of the Greater Omentum and Left Gastroepiploic Vascular Territory (Stations 4sb and 4d)

While the greater curvature was traditionally considered the standard starting point for gastric mobilization, the actual sequence of dissection steps depends on the surgeon’s assessment of tumor involvement in specific anatomical structures. Alternative approaches may include lesser omentum mobilization with left gastric vascular pedicle evaluation and diaphragmatic hiatus dissection for esophageal invasion assessment.

We preferred initial gastric mobilization without the greater omentum, using the following key landmarks: (1) vascular arcades along the greater curvature, (2) pyloric region, (3) the “crow’s foot” neural structure from Latarjet’s motor branch, (4) the anastomotic zone between right and inferior left gastric arteries—the watershed between lymphatic basins draining to stations 5 and 7 (Figure 6).

Moving 2–3 cm below the vascular arcade of great curvature to the gate of the spleen, we (1) determined the boundary of the lower pole and (2) performed a downward retraction of the splenic bend of the colon with visualization of the tail of the pancreas.

When dissecting the gastrocolic ligament rightward to its free edge, we deliberately avoided extending to the pylorus, as the mesogastric and mesocolic layers fuse above the pancreatic isthmus forming the third key landmark in embryologically guided gastrectomy where the right gastroepiploic and middle colic vessels converge.

With upward gastric traction, the left gastroepiploic vessels become identifiable at the splenic hilum. Reliable anatomical guides for locating the left gastroepiploic venous origin include the following: (1) superior border of pancreatic tail, (2) splenic inferior pole, (3) splenic flexure of colon.

**Figure 7:**
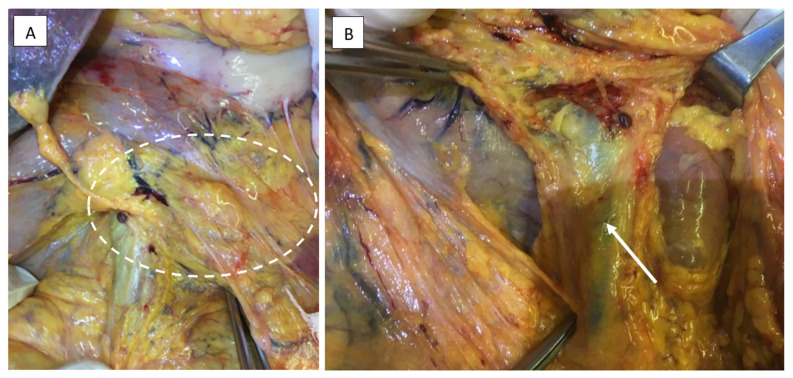
Steps of dissecting the mesogastric layer and the left gastroepiploic artery in a cadaveric specimen. (**A**) The mesogastric layer is outlined by a dashed line. (**B**) The left gastroepiploic artery is indicated by an arrow.

The vascular dissection proceeds through the avascular plane between the pancreatic tail and the splenic lower pole (Figure 7). In 8 cases, tumor infiltration into the greater or lesser omentum necessitated partial-to-complete omentectomy, consistent with reported oncologic principles in the literature [26].

#### 2.3.3. Lymphadenectomy of Stations 11p/11d and 10 (Splenic Artery and Hilar Nodes)

Key anatomical landmarks include the splenic artery and gastropancreatic ligament. Given the tumor’s location along the greater curvature, station 10 lymphadenectomy was performed in only two patients. In accordance with findings from the randomized JCOG 0101 trial, splenectomy was deliberately avoided [27]. When performing mediolateral lymphadenectomy in station 11, the recommended sequence is as follows: (1) initial identification of left gastric vessels at the gastropancreatic ligament root, (2) proximal vascular mobilization, (3) leftward dissection along the anterior surface of the splenic artery (visible at the pancreatic border), (4) tissue displacement toward the stomach, (5) termination point at the splenic artery’s ascending branch (posterior gastric artery origin). This ascending branch marks the lateral boundary for D2 lymphadenectomy during gastrectomy.

Continuing the lymphadenectomy along the superior and posterior contours of the splenic artery, we progressed retropancreatically until identifying the splenic vein—the posteroinferior boundary of D2 lymphadenectomy.

**Figure 8:**
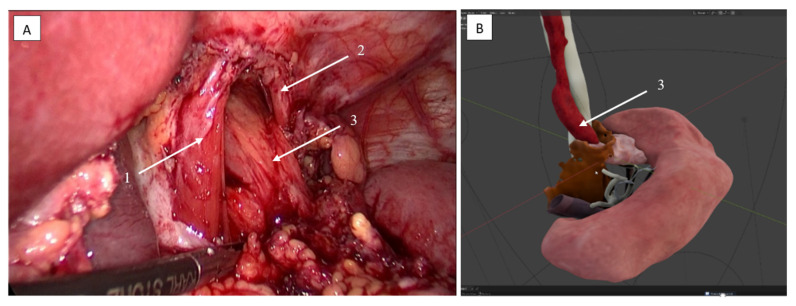
(**A**) Intraoperative photo of the cardioesophageal junction; (**B**) 3D model for VR technology application in preoperative navigation. Right crus of the diaphragm (1), left crus of the diaphragm (2), esophagus (3).

In this region, lateral to the left gastric vessels while advancing superiorly, we identified both medial diaphragmatic crura (Figure 8). The dissection base consisted of Gerota’s fascia with the underlying adrenal gland clearly visible.

This technique enables controlled vascular transection under direct vision—first of the anterior short gastric arteries, then the posterior gastric artery—while avoiding complete splenic devascularization or parenchymal injury. A critical refinement involves careful management of the left gastroepiploic artery: instead of immediate ligation at its origin, surgeons should meticulously trace its distal branches, which typically include (1) an inferior polar splenic branch (present in 42% of cases), (2) pancreatic tail collaterals via the pancreaticosplenic arcade, and (3) omental supply vessels when preserving the greater omentum. Division should occur ≥1.5 cm distal to the last tumor-involved branch, preserving perfusion via the preserved short gastrics and polar circulation. The anatomical safety net consists of intact ≥2 mm arterial inflow and unimpeded venous drainage through either short gastric or inferior polar veins.

To complete the lymphadenectomy in this area, we returned to the base of the posterior gastric artery, finally differentiating it from the upper pole splenic artery, which may originate proximal to the posterior gastric artery, and ligated it. The dissection proceeded along the upper semicircle of the splenic artery at the origin of the left gastroepiploic artery, which was fully mobilized at its base, where the omental branch and the lower pole splenic branch were identified, preserving the latter whenever possible. Subsequently, under direct visualization, the short gastric arteries were divided.

Thus, the splenic hilum was exposed, allowing for the removal of soft tissues in the hilar region. If necessary, the greater omentum could then be detached from the transverse colon from left to right without technical difficulty.

#### 2.3.4. Lymphadenectomy of Stations 4sb and 6 Was Performed

Key anatomical landmarks include the right and middle colic vessels, the round ligament of the liver, and the pylorus.

The mesocolon was mobilized, and the pylorus was identified and dissected free to the right of the pancreatic head up to the gastroduodenal artery. The root of the mesocolon and the hepatic flexure of the colon were retracted inferiorly to expose the right gastroepiploic vessels.

The right gastroepiploic vein was identified by tracing its confluence with the right colic vein, while the artery was located deeper and more medially. Proceeding distally along the gastroduodenal artery, the origin of the right gastroepiploic artery was identified, divided, and ligated at this level.

The right gastroepiploic vein runs slightly more lateral and superficial—closer to the greater curvature of the stomach—and is usually well-defined. To isolate its drainage into Henle’s trunk (the confluence with the right colic vein), it can be helpful to dissect along the mesocolon, moving from the hepatic flexure toward the left, following the right colic vein.

For safe navigation near the origin of the middle colic vessels, it is advisable to mobilize the hepatic flexure by separating it from the omentum and exposing the right semicircle of the duodenum. This improves anatomical orientation of the pancreatic head and adjacent structures. An additional landmark is the round ligament of the liver, lying in the same sagittal plane as the middle colic vessels. After transecting the right gastroepiploic vein, access to lymph node station 6 is achieved. Continuing the dissection laterally to the right, we proceeded to mobilize the mesocolon inferiorly from the pancreatic head toward the prepyloric vein—the lateral boundary of D2 lymphadenectomy. At this stage, the duodenal C-loop and the medially positioned pancreatic head served as key anatomical landmarks.

#### 2.3.5. Lymphadenectomy of Stations 5 (Suprapyloric) and 12a (Hepatoduodenal Ligament)

Prior to transecting the duodenum, we carefully developed a dissection plane posterior and superior to the duodenal bulb, then divided the duodenum. During this step, precise transection of the mesogastric layer was critical to avoid inadvertent dissection into the pancreatic isthmus. The gastroduodenal artery was then traced proximally to its origin from the common hepatic artery, allowing for definitive identification of the proper hepatic artery.

Lymph node dissection along the hepatoduodenal ligament (station 12a) was systematically performed in a distal-to-proximal direction, following the course of the proper hepatic artery toward the hepatic hilum. This approach was preferred over proximal-to-distal dissection due to its enhanced safety profile. The right gastric artery was deliberately preserved until the left hepatic artery branch was clearly visualized, ensuring anatomical certainty before vascular division.

The dissected tissues were gently retracted toward the lesser omentum to maintain exposure. Continuing cephalad along the left border of the proper hepatic artery to the hepatic hilum, the right gastric artery was identified and ligated at its origin. This maneuver allowed clear identification of the portal vein’s left border, a key anatomical landmark confirming adequate lymphadenectomy.

Throughout the procedure, we ensured preservation of at least one suprapyloric artery to maintain adequate vascular supply to the gastric remnant. This preservation was particularly focused on the anastomotic zone between the right and inferior left gastric arteries, which represents the watershed area between lymphatic basins draining to stations 5 and 7, while also protecting the “crow’s foot” neural structure derived from Latarjet’s motor branch.

The described technique strictly adhered to the natural boundaries of the mesogastric layer, with focus on three critical anatomical relationships: the fusion point at the pancreatic isthmus where mesogastric and mesocolic layers converge, the vascular watershed at the hepatoduodenal ligament, and the lymphatic transition zone between foregut and midgut derivatives. This anatomically precise approach allowed for consistent identification of the proper hepatic artery across all cases, complete avoidance of pancreatic isthmus injury, and successful preservation of collateral circulation to the gastric remnant in every procedure.

### 2.4. Clinical Outcomes

Using the developed system of topographic–anatomical landmarks, 22 gastrectomies with resection of the abdominal esophagus were performed, along with 39 Billroth I subtotal gastrectomies. Nine patients underwent Billroth II subtotal gastrectomy, and in eight additional cases, proximal gastrectomy was performed (Table 3).

The stage distribution according to TNM-8 classification was as follows:

- Stage IIIA (T2-4aN1-3M0): 16 patients (20.5%).
- Stage IIIB (T3-4bN0-3M0): 16 patients (20.5%).
- Stage IIIC (T4a-4bN2-3M0): 8 patients (10.3%).
- Stage IV (T × N × M1): 38 patients (48.7%).

Prior to the implementation of the topographic–anatomical navigation system for embryological plane resection, the mean duration of laparoscopic procedures was approximately 300 min. Following technical refinements to the methodology, the operative time decreased to approximately 250 min.

During the initial implementation phase of this technique, six conversions to open surgery were required: two cases (33.3%) due to iatrogenic injury of the proper hepatic artery, and four cases (66.7%) due to extensive tumor infiltration into the mesenteric root of the transverse colon preventing adequate mobilization.

Histopathological assessment demonstrated R0 resection (microscopically negative margins) in 74.4% of cases [16]. The remaining 25.6% of patients had R1 (20.5%) or R2 (5.1%) resections. All R1 resections with positive proximal margins (*n* = 20) were either palliative cytoreductive procedures (*n* = 16), performed as life-saving interventions with intentional margin compromise, or curative-intent resections (*n* = 4) for cases with pancreatic capsular invasion where more extensive surgery was precluded by severe comorbidities. Of note, the four R1 cases in the curative-intent group represented 5.1% of the total cohort.

One fatal outcome was recorded on the second day after laparoscopic gastrectomy due to pulmonary embolism (PE), despite ongoing anticoagulant therapy. Three other cases occurred on the 5th, 7th, and 8th days post-surgery, associated with progressive cardiopulmonary insufficiency, predominantly characterized by bilateral polysegmental pneumonia.

The complications observed were in patients from the older age group, which may be attributed to the severity of the primary disease (extent of the process, necessity of high esophageal transection due to tumor localization), and subcompensated or decompensated chronic comorbidities. Perioperative outcomes are detailed in Table 4.

A comparative analysis of intraoperative outcomes in the main group and the comparison group, without 3D model construction, is presented in Table 5.

### 2.5. Artificial Intelligence (AI) Statement

Text proofreading and stylistic refinement were performed using the DeepSeek-V3 AI assistant. The tool was exclusively used for linguistic improvements, with all substantive content modifications made by human authors.

## 3. Results

Table 5 presents the results of the comparative analysis of patient groups with and without preoperative 3D planning. The applied statistical methods and corresponding *p*-values are indicated. The Mann–Whitney U test was used for quantitative variables, and Fisher’s exact test was used for categorical variables. Statistically significant differences were found in the number of removed lymph nodes, mean duration of surgery, and radicality of surgical intervention.

**Figure 9:**
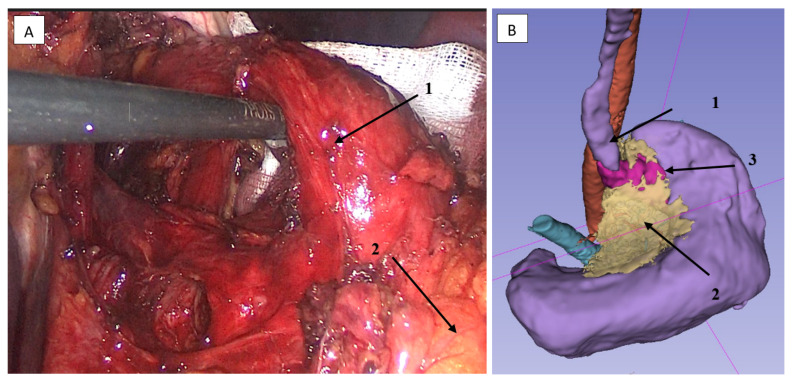
(**A**) Intraoperative photo showing the mobilization stage of the cardioesophageal junction area; (**B**) 3D reconstruction of the stomach with surrounding structures and tumor. Esophagus (1), mesogastric layer (2), tumor (3).

**Figure 10:**
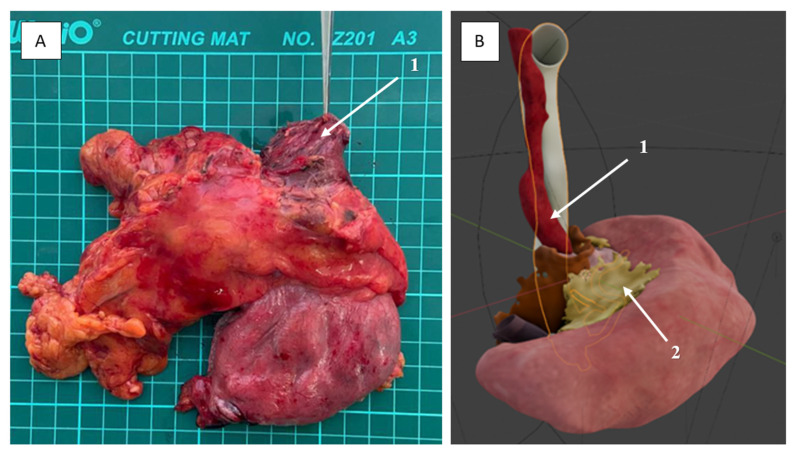
(**A**) Photo of the resected gross specimen; (**B**) preoperative 3D model for VR technologies. Esophagus (1), mesogastric layer (2).

The statistical analysis revealed significant differences between the groups of patients who underwent preoperative 3D planning and those who did not. The most pronounced difference was observed in the number of removed lymph nodes: in the 3D planning group, this parameter was statistically significantly higher (Mann–Whitney U test: *p* < 0.00001), which may indicate more thorough lymph node dissection control when using spatial modeling technologies. Additionally, it was established that the mean duration of surgery in this group was longer (*p* = 0.047), which may be associated with a more complex surgical technique guided by the 3D model or a more radical approach to the removal of affected structures (Figure 9 and Figure 10).

For variables such as conversion to laparotomy (*p* = 0.070) and postoperative mortality (*p* = 0.189), no statistically significant differences were identified. However, the trend toward fewer conversions with the use of 3D planning remains clinically relevant and warrants further validation in larger cohorts.

The assessment of differences in variability (variance) of quantitative parameters between groups using the Brown–Forsythe test did not reveal statistically significant differences (e.g., variance in the number of lymph nodes: *p* = 0.564). The relatively low variance may be attributed to the limited sample size and the deliberate exclusion of outlier clinical cases from the statistical analysis, as their number was insufficient to form a distinct subgroup at this stage. This exclusion was driven by the need to establish a homogeneous dataset for the initial evaluation of 3D planning applicability and may be reconsidered as more clinical data are accumulated.

Analysis of the categorical variable demonstrated that achieving R0 radicality was statistically significantly more frequent in patients who underwent 3D planning (*p* = 0.045, Fisher’s exact test), suggesting a potential role of 3D visualization technologies in enhancing the oncological radicality of surgical interventions.

Preoperative 3D modeling of the mesogastric layer in some patients enabled the identification of potential tumor involvement in areas with higher densitometric density. Obtaining such data at the preoperative stage enhances understanding of the mesogastric layer’s characteristics and facilitates more detailed surgical planning, adhering to the principles of safe surgery.

This approach appears promising and warrants further investigation. The development of the laparoscopic technique with the establishment of a topographic–anatomical navigation system was conducted from 2012 to 2014 (26 patients). The outcomes of surgical treatment improved with its subsequent application from 2015 to 2018 (52 patients).

The implementation of the developed topographic–anatomical navigation methodology expanded the capabilities of laparoscopic access in cases of complicated gastric cancer.

## 4. Discussion

In the surgical management of malignant neoplasms of the gastrointestinal tract, it is generally recommended to resect the tumor-bearing organ along with its mesentery [2]. Recent advancements in minimally invasive surgery have led to the predominance of laparoscopic procedures, with gastrectomy performed within the embryonic mesogastric layer emerging as the gold standard in oncological surgery.

Mesogastrectomy would be straightforward if the stomach and intestines formed a straight tube aligned along a single plane [2]. However, embryonic processes such as intestinal tube rotation and mesenteric spiraling generate multidirectional planar vectors, complicating the surgeon’s task.

A major obstacle in performing mesogastrectomy is the dissection of fused embryonic mesenteric layers at critical points, necessitated by both technical requirements and oncological appropriateness.

High-quality global data demonstrate satisfactory treatment outcomes for complicated gastric cancer, encompassing both early-stage and locally advanced cases [28,29,30,31,32]. As of today, the trajectory of gastric cancer surgery is focused on minimizing surgical aggressiveness while preserving the oncological principles of the intervention [19,20,21,33,34].

As early as 1982, R. Heald, followed by other authors, demonstrated that tumor resection within the mesorectal layer enables local tumor control and reduces the recurrence rate [6,7,23,34,35].

The safety of operating within the embryonic layer is determined by the absence of major vessels and is well illustrated by the standards long established by R. Heald: (1) recognition of mobility between tissues of different embryonic origins; (2) sharp dissection under direct visual control with adequate lighting; (3) meticulous (tear-free) exposure of the dissection plane through controlled traction and countertraction [23].

During gastrectomy, we were compelled to transect the embryonic layer twice (at the pancreatic isthmus and at the splenic hilum) to adequately advance in the retroperitoneal space.

The mesogastric layer terminates to the left beyond the left gastroepiploic artery (group 10 lymph nodes). Embryologically, this region pertains to the dorsal mesentery, from which the spleen originates. However, due to rotation and fusion of the mesentery, this area primarily serves as a regional lymphatic drainage pathway for the spleen rather than the stomach and, in fact, does not constitute the mesogastrium. This likely explains why metastatic involvement of the splenic hilum is less frequent compared to other lymph node groups. Consequently, it has been excluded from standard D2 lymphadenectomy, except in specifically indicated cases (e.g., direct tumor invasion).

The variability in the anatomy of the splenic artery and its branches, particularly in the context of a mesogastric layer compromised by locally advanced disease, necessitates their precise intraoperative identification to prevent complications such as ischemia or infarction of the upper or lower pole of the spleen, or injury to its capsule. For instance, prior to ligating the so-called posterior gastric artery, it is essential to trace it to its origin to avoid inadvertently ligating a branch directly supplying the upper pole of the spleen. Similarly, at the splenic hilum, this artery must be differentiated from the left gastroepiploic artery. A comparable approach is recommended before ligating the left gastroepiploic artery at the level of its bifurcation with the branch supplying the lower pole of the spleen [2,36,37].

Navigation along the splenic artery warrants separate description due to its frequently tortuous course, which increases the risk of injury. During lymphadenectomy within the embryonic layer, we removed not only lymph nodes but also adipose tissue within the space corresponding to the embryonic dorsal mesentery. Transection of this layer was performed only when necessary to preserve the pancreas and spleen, which are embryologically situated within the same mesenchymal layer as the stomach. The indications for removing specific lymph node groups depended on the extent of the tumor process.

Manipulations in the retroperitoneal layer, within the loose adipose spaces of the mesogastric layer, without three-dimensional imaging or tactile feedback, prompted us to develop and describe a system of visual landmarks—consistently identifiable anatomical structures—enabling safe transitions between landmarks.

In our study, 3D models of perigastric adipose tissue (mesogastric layer) were generated based on differences in densitometric densities. The primary hypothesis for defining the boundaries of the affected mesolayer relied on detecting variations in densitometric density between normal and affected adipose tissue (resulting from lymphatic edema, enlarged lymph nodes, or tumor deposits) following contrast enhancement (87 ± 23 HU vs. 110 ± 25 HU). This approach may potentially enable the identification of affected lymph node groups and provide a clear visualization of the extent of involvement through a 3D model.

Despite the lack of data on long-term outcomes of mandatory surgeries for gastric cancer [28,38,39,40], cytoreductive surgical interventions combined with systemic chemotherapy in patients with complicated locally advanced gastric cancer may provide prolonged survival [41].

Standardization of the embryologically oriented gastrectomy technique reduces the likelihood of conversions and other complications while preserving the principles of oncological radicality.

The integration of Internet of Things (IoT) technologies represents a paradigm shift in modern surgery. As highlighted, IoT-enabled devices enhance intraoperative navigation by providing real-time anatomical guidance during complex procedures like laparoscopic gastrectomy—critical for oncological precision in mesogastric dissection. Concurrently, IoT-driven data aggregation fuels AI algorithms for preoperative 3D modeling (e.g., tumor reconstruction) and predictive analytics, mitigating risks such as vascular injury or incomplete resection [42]. This synergy aligns with the study’s emphasis on embryological plane accuracy and standardized landmarks.

## 5. Conclusions

The conducted study aimed to formulate an algorithm for manipulations within the altered mesogastric layer and to standardize this methodology, taking into account global experience in anatomical research and contemporary trends in embryologically oriented gastric cancer surgery.

The authors endeavored to articulate a sequence of actions, replacing the concept of “finding” with “visualizing” clear landmarks, even in the context of a mesolayer compromised by the oncological process.

Currently, such studies are rare, but given the trends in research, they are highly sought after. The intraoperative topographic–anatomical navigation system, developed and integrated into routine clinical practice at our clinic for endoscopic surgical treatment of complicated forms of gastric cancer, has enabled the standardization of surgical techniques regardless of the extent of the tumor process and optimized treatment outcomes in this patient group.

The recommended topographic–anatomical navigation landmarks, grounded in an understanding of intestinal tube embryogenesis and key anatomical reference points, while considering the concept of embryonic dissection planes, have facilitated the safe performance of necessary endoscopic surgical interventions for complicated locally advanced gastric cancer of various localizations.

## Figures and Tables

**Table 1:** Clinical characteristics of patients.

	*N* = 78 (%)
Mean age, years (confidence interval—CI)	60 (58–62)
**Gender Distribution**	*n* (%)
Male	53 (67.9)
Female	25 (32.1)
**ASA Physical Status**	*n* (%)
ASA I	15 (19.2)
ASA II	22 (28.2)
ASA III	41 (52.6)
**ECOG Performance Status**	*n* (%)
0	11 (14.1)
1	52 (66.7)
2	15 (19.2)
**Comorbidity Distribution**	*n* (%)
Cardiovascular comorbidities (hypertension, coronary artery disease) stratified using NYHA guidelines	72 (92.3)
Type 2 diabetes mellitus	20 (25.6)
Peptic ulcer disease (gastric and duodenal ulcers)	9 (11.5)
Chronic obstructive pulmonary disease	7 (9.0)
**Mean BMI (CI)**	27.1 (23.4–33.2)
**Tumor Localization**	*n* (%)
Gastric cardia	4 (5.1)
Gastric body	4 (5.1)
Antrum/pyloric region	48 (61.5)
Subtotal involvement	15 (19.2)
Total gastric involvement	7 (9.0)
**Life-Threatening Complications Requiring Emergency Intervention**	*n* (%)
Bleeding	22 (28.2)
Stenosis	41 (52.6)
Combined gastric outlet obstruction and recurrent bleeding	9 (11.5)

**Table 2:** Delineation of one of the slices of the affected mesogastric layer boundaries based on the difference in densitometric perigastric adipose tissue density.

Densitometric Density Points	Mesogastric Layer Along the Left Gastric Artery (HU)	Mesogastric Layer in the Region of Retrogastric Tissue (HU)
1	−88.2	−28.2
2	−78.8	−26.6
3	−92.9	−27.7

**Table 3:** Surgical procedures performed and local tumor invasion patterns (*N* = 78).

Surgical Procedure		Cases, *n* (%)
Total gastrectomy with abdominal esophageal resection		22 (28.2%)
Distal subtotal gastrectomy	Billroth I reconstruction	39 (50.0%)
	Billroth II reconstruction	9 (11.5%)
Proximal gastrectomy with abdominal and lower thoracic esophageal resection		8 (10.3%)
**Locally Invaded Structures**		**Cases, *n* (%)**
Abdominal esophagus		11 (14.1%)
Greater/lesser omentum		46 (59.0%)
Pancreatic capsule		4 (5.1%)
Splenic hilum (direct invasion)		4 (5.1%)
Duodenum		7 (9.0%)

**Table 4:** Perioperative outcomes and histopathology.

Indicator	Number of Observations (*N* = 78), *n* (%)
Mean duration of surgery, min (CI)	259 (251–266)
Mean intraoperative blood loss, mL (CI)	185 (177–192)
Number of conversions to laparotomy, *n* (%)	6 (7.7)
Mean duration of ICU stay, days (CI)	2 (1–2)
Mean duration of narcotic analgesia, days (CI)	2 (2–3)
Mean hospital stay, days (CI)	8 (7–9)
Total number of postoperative complications, *n* (%)	28 (35.9)
Postoperative mortality, *n* (%)	4 (5.1)
Mean number of removed lymph nodes (CI)	22 (21–22)
*Radicality, n (%)*	
R0	58 (74.4)
R1	16 (20.5)
R2	4 (5.1)
*Tumor type, n (%)*	
Adenocarcinoma	65 (83.3)
Signet ring cell carcinoma	13 (16.7)
*Degree of histological differentiation, n (%)*	
High grade (G1)	9 (11.5)
Moderate grade (G2)	14 (17.9)
Low grade (G3)	33 (42.3)
Undifferentiated carcinoma (G4)	22 (28.2)
*TNM stage, n (%)*	
IIIA	16 (20.5)
IIIB	16 (20.5)
IIIC IV	8 (10.3) 38 (48.7)

**Table 5:** Results of statistical analysis of differences between groups with and without 3D planning.

Indicator	Method	Tested Characteristic	*p*-Value
Duration of surgery	Mann–Whitney U test	Difference between groups	0.047
Conversion to laparotomy			0.070
Postoperative mortality			0.189
Number of removed lymph nodes			<0.00001
Duration of surgery	Brown–Forsythe test	Difference in variances	0.365
Conversion to laparotomy			0.068
Postoperative mortality			0.187
Number of removed lymph nodes			0.564
Radicality R0 vs. R1	Fisher’s exact test	Categorical association	0.045

## Data Availability

The original data presented in this study are included in the article. Further inquiries can be directed to the corresponding author.

## Abbreviations

3D	Three-Dimensional
ASA	American Society of Anesthesiologists
BMI	Body Mass Index
CI	Confidence Interval
CT	Computed Tomography
DICOM	Digital Imaging and Communications in Medicine
ECOG	Eastern Cooperative Oncology Group
G1/G2/G3/G4	Histological Grade (differentiation)
HU	Hounsfield Units
ICG	Indocyanine Green
ICU	Intensive Care Unit
JCOG	Japan Clinical Oncology Group
KLASS	Korean Laparoendoscopic Gastrointestinal Surgery Study Group
NCCN	National Comprehensive Cancer Network
NYHA	New York Heart Association
PE	Pulmonary Embolism
R0/R1/R2	Resection Margins (R0: no residual tumor, R1: microscopic residual, R2: macroscopic residual)
TNM	Tumor, Node, Metastasis (staging system)
VR	Virtual Reality
